# Stearoyl-CoA desaturase 1 integrates tissue-specific oncogenic pathways into a pan-cancer ferroptosis resistance program

**DOI:** 10.1038/s41419-026-08982-w

**Published:** 2026-06-19

**Authors:** Francesca Ascenzi, Giovanni Blandino, Gennaro Ciliberto, Rita Mancini

**Affiliations:** 1 Translational Oncology Research Unit, Department of Research, Diagnosis and Innovative Technologies, IRCCS Regina Elena National Cancer Institute, Rome, Italy; 2 Department of Clinical and Molecular Medicine, Sapienza University of Rome, Rome, Italy; 3 Takis s.r.l., 00128 Rome, Italy; 4 Scuola Superiore di Studi Avanzati Sapienza (SSAS), Sapienza University of Rome, Rome, Italy

**Keywords:** Cancer therapy, Cell death

## Abstract

Ferroptosis, a regulated form of cell death driven by iron-dependent lipid peroxidation, has recently emerged as a promising therapeutic vulnerability in cancer. Among its key modulators, stearoyl-CoA desaturase 1 (SCD1) plays a pivotal role in ferroptosis resistance by converting saturated fatty acids into monounsaturated fatty acids (MUFAs), thereby limiting the accumulation of highly peroxidizable polyunsaturated fatty acids (PUFAs) and stabilizing membrane integrity under oxidative stress. Multiple oncogenic, transcriptional, epigenetic, and microenvironmental cues enhance tumor survival by promoting SCD1-dependent ferroptosis resistance. Despite this diversity of regulatory inputs, a unifying principle emerges across tumor types: SCD1 activity preserves lipid desaturation as a dominant metabolic strategy to suppress ferroptotic cell death. In this review, we critically analyze how SCD1-dependent ferroptosis resistance is shaped by tumor-specific metabolic states, microenvironmental pressures, and regulatory hierarchies across multiple malignancies. We identify recurring mechanistic themes through which SCD1 integrates redox control, lipid metabolism, stemness, and therapy resistance, while highlighting how these processes are differentially regulated across tissues. Preclinical evidence indicates that targeting SCD1, particularly in rational combination with ferroptosis inducers, chemotherapy, radiotherapy, or immunotherapy, can lower the ferroptotic threshold and overcomes treatment resistance. Finally, we discuss translational challenges and emerging strategies, including tumor-selective delivery, adaptive dosing, and context-specific combinations, that may enable safe and effective exploitation of the SCD1-ferroptosis axis in cancer therapy.

## Facts

- SCD1 is a key metabolic enzyme that converts saturated fatty acids into monounsaturated fatty acids, limiting lipid peroxidation and protecting tumor cells from ferroptosis.
- Elevated SCD1 expression is recurrent across multiple tumor types, but arises through distinct tissue-specific oncogenic, transcriptional, and microenvironmental mechanisms.
- Tumor metabolic context and microenvironmental features, such as oxygen tension, lipid availability, immune-derived signals, and nutrient flux, critically shape how SCD1 supports ferroptosis resistance in different cancers.
- Pharmacological or genetic disruption of SCD1 activity consistently increases lipid peroxidation and sensitizes tumor cells to ferroptosis, often enhancing the efficacy of chemotherapy, radiotherapy, or targeted therapies.

## Open questions

- Which tumor-specific metabolic states and microenvironmental contexts define dependency on SCD1-mediated ferroptosis resistance, and how can these be exploited for patient stratification?
- Can context-adapted therapeutic strategies, including intermittent dosing or tumor-activated delivery systems, selectively target SCD1 while minimizing systemic toxicity?
- What are the most effective combination strategies integrating SCD1 inhibition with ferroptosis inducers, chemotherapy, radiotherapy, or immunotherapy in different tumor settings?

## Introduction

Ferroptosis is a distinct form of regulated cell death first described in 2012, characterized by the accumulation of iron-dependent lipid reactive oxygen species [1]. Unlike apoptosis or necroptosis, ferroptosis does not rely on caspase activation or receptor-interacting protein kinase (RIPK) signaling. Instead, it is driven by the failure of cellular antioxidant systems to counteract iron-catalyzed lipid peroxidation, particularly of polyunsaturated fatty acid (PUFA)-containing phospholipids within cellular membranes.

**Fig. 1:**
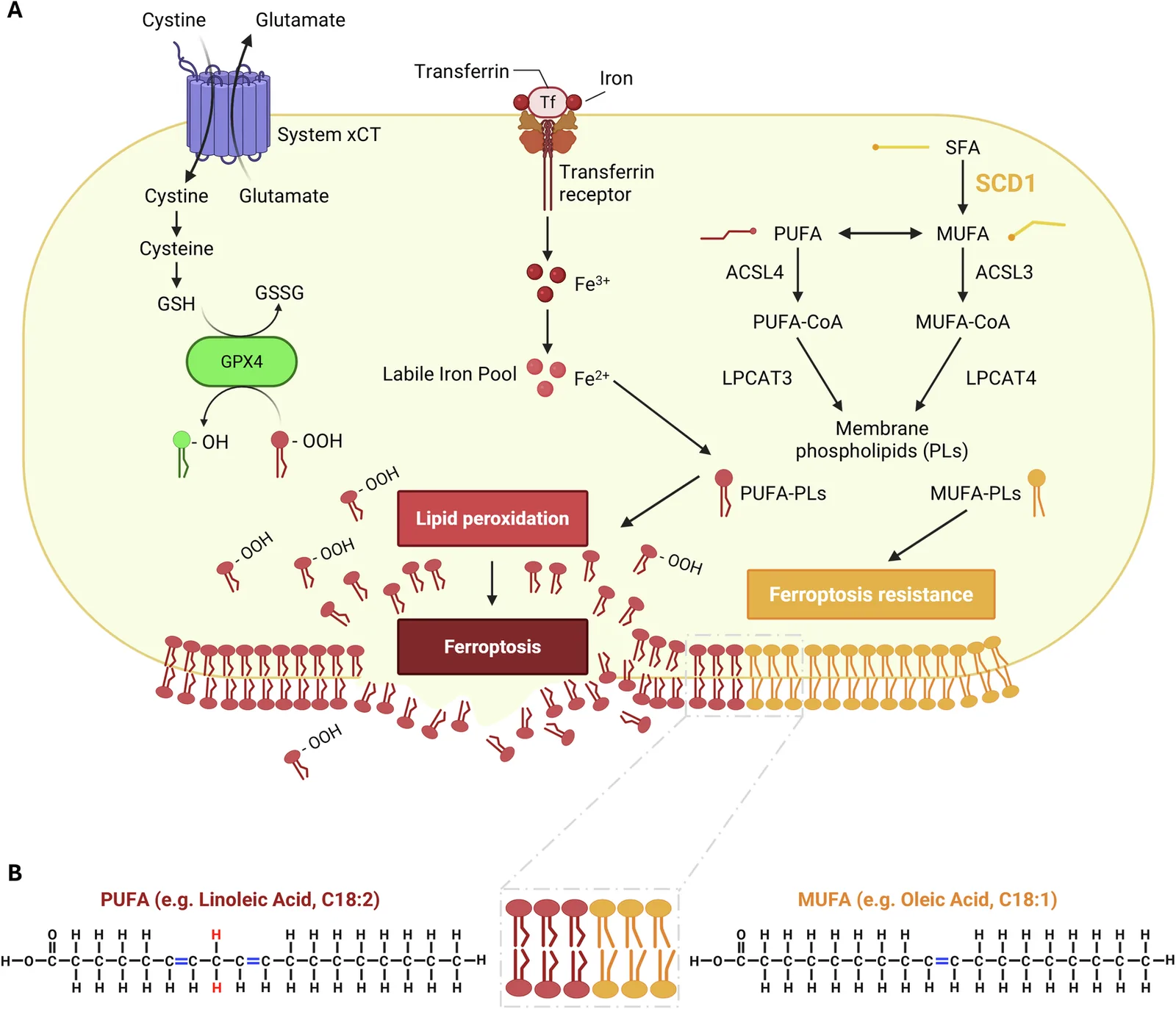
SCD1-dependent lipid remodeling and ferroptosis resistance. **A** Schematic overview of the main molecular players regulating ferroptosis in cancer. Iron metabolism provides the catalytic center for ROS generation via the Fenton reaction, driving lipid peroxidation. Antioxidant defense relies on GPX4, which detoxifies lipid hydroperoxides using glutathione (GSH) supplied by cysteine import through System Xc⁻. Lipid metabolism enzymes, including ACSL4 and LPCAT3, promote the incorporation of polyunsaturated fatty acids (PUFAs) into membrane phospholipids, sensitizing them to peroxidation. In contrast, the SCD1-ACSL3-LPCAT4 axis favors monounsaturated fatty acid (MUFA) synthesis and incorporation, thereby stabilizing membrane lipids and conferring resistance to ferroptosis. **B** Structural basis for the differential susceptibility of PUFAs and MUFAs to lipid peroxidation. Double bonds are highlighted in blue, while bis-allylic hydrogens are indicated in red, marking the most reactive sites in PUFAs that initiate peroxidation chain reactions. Their absence in MUFAs confers higher chemical stability and protection against ferroptosis. Created in BioRender. Ascenzi, F. (2026) https://BioRender.com/ij5uv6w.

The molecular machinery governing ferroptosis encompasses several key components [2]. Iron metabolism plays a central role, as labile iron catalyzes the Fenton reaction that generates reactive oxygen species capable of initiating lipid peroxidation. Ferroptosis is modulated by specialized proteins involved in lipid metabolism, notably acyl-CoA synthetase long-chain family member 4 (ACSL4) and lysophosphatidylcholine acyltransferase 3 (LPCAT3). ACSL4 catalyzes the ligation of free polyunsaturated fatty acids (PUFAs) with coenzyme A, generating PUFA-CoA derivatives that serve as substrates for phospholipid biosynthesis. LPCAT3 subsequently incorporates these activated fatty acids into membrane phospholipids, thereby rendering them particularly susceptible to peroxidation (Fig. 1A). Glutathione peroxidase 4 (GPX4) serves as the primary guardian against ferroptosis, catalyzing the reduction of lipid hydroperoxides to their corresponding alcohols using glutathione as a cofactor. System Xc-, a cystine/glutamate antiporter, supplies cysteine for glutathione synthesis, forming another critical node in the ferroptosis defense network.

Due to its dependency on redox imbalance and metabolic stress, ferroptosis represents a therapeutic vulnerability in cancer [3–5]. However, tumor cells often adapt by rewiring lipid metabolism to evade this lethal pathway. Among these adaptations, stearoyl-CoA desaturase 1 (SCD1) has emerged as a key modulator [6, 7]. By converting saturated fatty acids (SFAs) into monounsaturated fatty acids (MUFAs), SCD1 reshapes membrane composition to resist peroxidation and maintain redox homeostasis. In particular, SCD1 activity promotes the enrichment of MUFAs within membrane phospholipids at the expense of polyunsaturated fatty acids (PUFAs), thereby limiting the availability of oxidation-prone lipids that fuel ferroptotic cell death.

Recent reviews have provided important conceptual frameworks for understanding SCD1 as a central regulator of lipid metabolism, cellular stress responses, and ferroptosis across cancer and non-cancer contexts [7, 8]. In this review, we examine how tissue-specific metabolic and microenvironmental contexts influence the regulation and functional relevance of SCD1 in ferroptosis resistance across tumor types.

## SCD1: a central regulator of lipid metabolism and ferroptosis resistance

SCD1 is an endoplasmic reticulum-resident, iron-containing enzyme that catalyzes the Δ9-desaturation of SFAs, such as primarily palmitic acid (C16:0) and stearic acid (C18:0), converting them into the MUFAs palmitoleic acid (C16:1) and oleic acid (C18:1), respectively [9]. This reaction represents a rate-limiting step in MUFA biosynthesis and profoundly alters the biophysical properties of fatty acids by lowering their melting point and increasing their conformational flexibility [9]. Following MUFA synthesis by SCD1, additional enzymes ensure their effective incorporation into membrane phospholipids. Long-chain acyl-CoA synthetases such as ACSL3 activate MUFAs by converting them into their CoA derivatives, while acyltransferases like LPCAT4 facilitate their insertion into phospholipids (Fig. 1A).

Critically, by elevating the MUFA content in cellular membranes, SCD1 reduces their susceptibility to lipid peroxidation and confers resistance to ferroptosis. This protective effect is rooted in the distinct chemical structures of PUFAs and MUFAs. PUFAs contain bis-allylic hydrogens, which are hydrogens bound to carbon atoms situated between two double bonds. These positions are chemically weak points: when a free radical abstracts a bis-allylic hydrogen, the unpaired electron that remains is stabilized by resonance with the adjacent double bonds. This resonance stabilization greatly increases the likelihood of hydrogen abstraction, making bis-allylic sites the primary entry points for initiating the lipid peroxidation chain reaction [10]. In contrast, MUFAs possess only a single double bond and therefore lack bis-allylic hydrogens. Without these vulnerable sites, MUFAs are chemically more stable and less prone to oxidative damage, resulting in membranes that are more resistant to ferroptosis (Fig. 1B).

Functional evidence supports the role for SCD1-driven MUFA synthesis in limiting lipid peroxidation and ferroptosis. Endogenous SCD1 activity has been shown to alter phospholipid composition [11], and increased MUFA availability can be incorporated into phospholipids to reduce ferroptotic sensitivity [12]. These observations support a link between SCD1 activity, membrane lipid composition, and ferroptosis resistance, while the precise mechanisms governing MUFA partitioning into specific ferroptosis-relevant lipid pools remain incompletely defined.

**Fig. 2:**
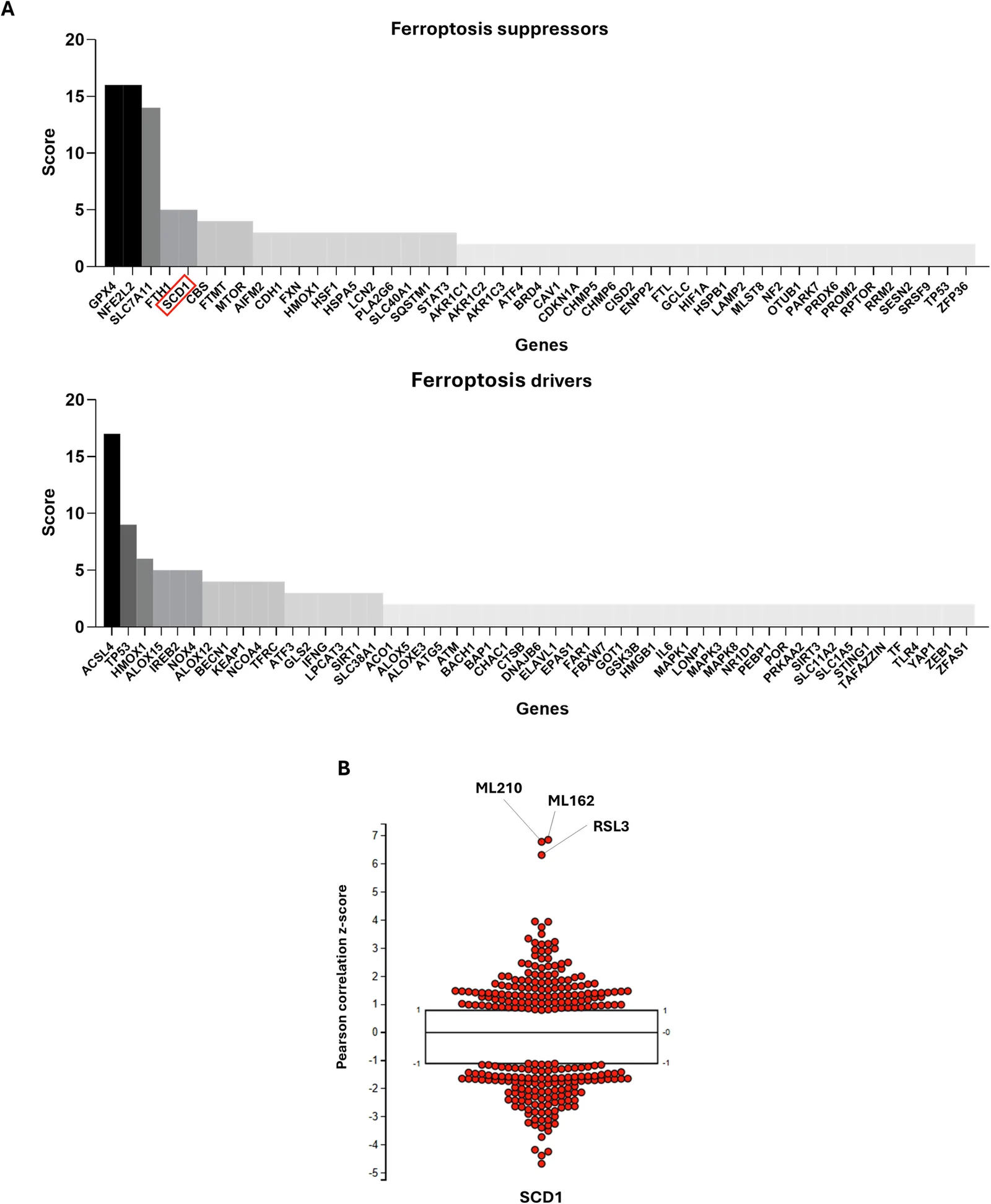
SCD1 is a ferroptosis suppressor gene. **A** Bar plots showing the main ferroptosis suppressors and drivers reported in the FerrDb database. The score represents the number of studies in which each gene has been identified with the corresponding role. SCD1 is highlighted with a red box. **B** Box-and-whisker plots display compounds (red dots) for which SCD1 expression levels are correlated with cell line sensitivity in CTRP. Values represent Z-scored Pearson’s correlation coefficients. A positive z-score indicates that higher SCD1 expression is associated with increased resistance to compounds, whereas a negative z-score reflects increased sensitivity. For the ferroptosis inducers ML-162, ML-210, and RSL3, the z-scores are 6.86, 6.79, and 6.32, respectively.

According to FerrDb, a curated database of ferroptosis regulators, SCD1 is annotated as a suppressor of ferroptosis [13]. This classification reflects the consistent association of SCD1 activity with reduced ferroptotic responses across independent studies, positioning SCD1 it alongside canonical ferroptosis defense nodes such as GPX4, NFE2L2, SLC7A11, and FTH1 (Fig. 2A). To complement this curated information with an orthogonal approach, we performed an analysis of the Cancer Therapeutics Response Portal (CTRP) datasets, assessing associations between gene expression profiles across 842 cancer cell lines and their sensitivities to 266 compounds [14]. Our results (Fig. 2B) revealed that SCD1 transcript levels correlate with relative resistance to ferroptosis inducers, with the strongest correlations observed for ML-162, ML-210, and RSL3.

The regulation of SCD1 expression and activity is intricate and multi-layered. At the transcriptional level, SCD1 is controlled by various factors including sterol regulatory element-binding proteins (SREBPs), liver X receptors (LXRs), peroxisome proliferator-activated receptors (PPARs), and carbohydrate-responsive element-binding protein (ChREBP) [15]. These transcription factors respond to nutritional and hormonal cues, including insulin, glucose, fatty acids, cholesterol, and inflammatory mediators [15]. Post-transcriptional regulation involves microRNAs and RNA-binding proteins that modulate SCD1 mRNA stability and translation efficiency. Post-translationally, SCD1 undergoes rapid turnover through ubiquitin-proteasome-mediated degradation, providing another layer of activity control.

In the context of cancer, SCD1 is frequently upregulated through various mechanisms, including oncogenic signaling, loss of tumor suppressors, hypoxia-induced responses, and alterations in the tumor microenvironment [16, 17]. Elevated SCD1 activity contributes to several hallmarks of cancer, including sustained proliferation, evasion of apoptosis, and metabolic rewiring [7, 16, 17].

## Cancer-specific roles and mechanisms in ferroptosis regulation

Evidence supporting the role of SCD1 in ferroptosis resistance derives from heterogeneous experimental approaches, ranging from correlative omics analyses to functional genetic and pharmacological perturbations.

The involvement of SCD1 in cancer biology reveals distinct patterns across different malignancies, reflecting tissue-specific metabolic constraints and microenvironmental pressures. In this section, tumor types are discussed according to their tissue of origin and physiological context. The sequence of tumor entities has been defined based on three main criteria: the availability of mechanistic evidence linking SCD1 to ferroptosis regulation, the representation of distinct metabolic and microenvironmental conditions, and the clinical relevance of SCD1 targeting. This organization allows a comparative view of how diverse metabolic adaptations and regulatory mechanisms converge on SCD1 to suppress ferroptosis across cancer types.

**Fig. 3:**
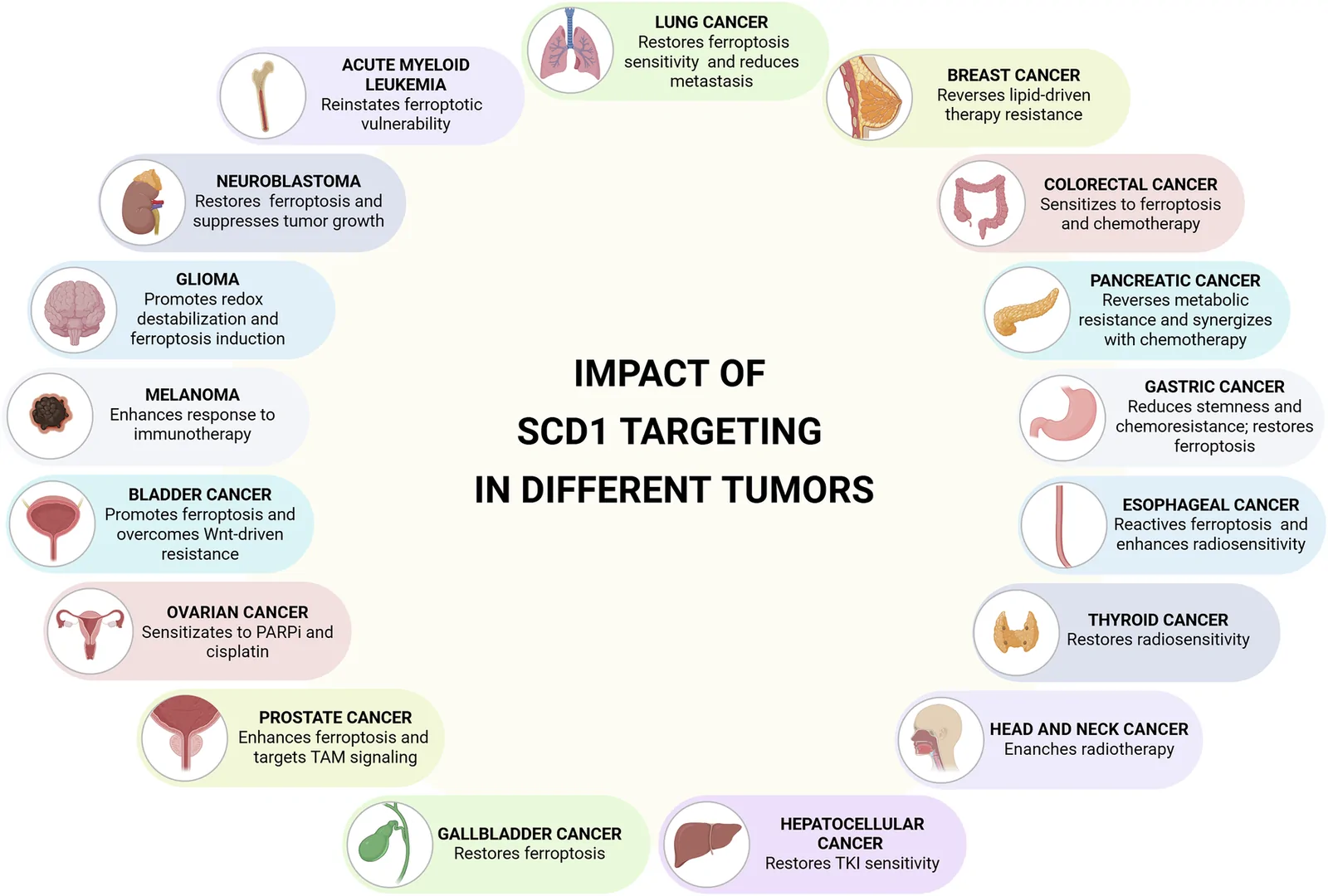
Therapeutic implications of SCD1 targeting across malignancies. Created in BioRender. Ascenzi, F. (2026) https://BioRender.com/3nta4gb.

Despite the heterogeneity of upstream pathways, SCD1 consistently emerges as a conserved metabolic node that protects cancer cells from lipid peroxidation and ferroptotic cell death. Key regulatory pathways and tumor-specific mechanisms are summarized in Table 1 and Fig. 3.

**Table 1:** Summary of SCD1 regulation and ferroptosis resistance mechanisms across cancer types.

Cancer type	Key regulatory pathways	Modulators of SCD1	Ferroptosis resistance mechanisms	Therapeutic implications	References
**Lung**	AKT/GSK3β/NRF2, PI3K/AKT/mTOR–SREBP1, LSH–EGLN1–c-Myc, EMT/Zeb1, STAT3 (microglia)	STK11/KEAP1 co-mutations, GPER1, LSH, FADS2, ZEB1, tumor-microglia signaling	MUFA synthesis, lipid ROS buffering, metabolic/epigenetic remodeling, microenvironmental support	SCD1 inhibition enhances ferroptosis sensitivity, disrupts metabolic adaptation, reduces stem-like properties, and limits metastatic progression	[18–29]
**Breast**	PI3K/AKT/mTOR–SREBP1, CB1 signaling, circSCD1–OTUB1 axis	FABP4, FADS1/2, CB1, circSCD1, SREBP1, PTEN loss ADAR1/2, PNKP	MUFA synthesis, lipid uptake from stroma, protection from lipid peroxidation, stabilization of SCD1 protein, ferritinophagy regulation	SCD1 inhibition or targeting its regulators (FABP4, FADS1/2, CB1, circSCD1) restores ferroptosis sensitivity, suppresses tumor growth, and sensitizes to chemotherapy	[30–39]
**Colorectal**	TIGAR–GSH, Smad2/3–SCD1, PI3K/AKT/mTOR–SREBP1, Hippo/YAP–Nrf2, p38/SCD1, AMPK–SCD1	TIGAR, Nodal, circRHBDD1, LINC01606, GPR37, PPP2CA, SLC38A5, DHX33, MYLK4, PIK3CA mutations	MUFA synthesis, mRNA stabilization, oxidative stress buffering, p53 repression relief, membrane protection	SCD1 inhibition or suppression of its upstream regulators (TIGAR, Nodal, PI3K/mTOR, GPR37) enhances ferroptosis sensitivity and improves response to therapy (e.g., aspirin combination)	[40–49]
**Pancreatic**	FBW7/NR4A1, MEK/ERK, BHLHE40/SREBP1/SCD1, NCOA6/SREBP1, mTOR/SREBP1/SCD1, MEN1/Mtor, CD147–SREBP1	FBW7, BHLHE40, NCOA6, SLC38A5, ZNF488, MEN1, CD147	MUFA synthesis, lipid membrane stabilization, chemoresistance	SCD1 inhibition or restoration of FBW7 sensitizes to ferroptosis and gemcitabine; mTOR/SREBP1/SCD1 blockade enhances therapy	[50–56]
**Gastric**	SCD1/Wnt/β-catenin, SCD1/SQLE/cholesterol/mTOR, exo-lncFERO/hnRNPA1/SCD1	Wnt/β-catenin, SQLE, lncFERO, hnRNPA1, p53, TPM4	MUFA and cholesterol synthesis, CSC maintenance, ferroptosis suppression	SCD1 inhibition or targeting Wnt/β-catenin and lncFERO axes induces ferroptosis and reduces stemness	[57–61]
**Esophageal**	Wnt/β-catenin/SCD1, BACH1–SCD1–OA, AMPK/SREBP1/SCD1	BACH1, zinc deficiency (ZD), p-AMPK	MUFA synthesis under ZD, OA-mediated protection, lipid peroxidation control	SCD1 inhibition enhances radiosensitivity; targeting ZD/SREBP1/SCD1 restores ferroptosis	[62–64]
**Thyroid**	YTHDF2–m6A–SREBF1–SCD1	YTHDF2	Epitranscriptomic SCD1 upregulation, radioresistance	YTHDF2 targeting restores radiosensitivity	[65]
**Head and Neck**	DDR1–Akt/mTORC1–SREBP1–SCD1	DDR1, acidic microenvironment	Acid niche–dependent MUFA synthesis	SCD1 inhibition enhances radiotherapy	[66, 67]
**Hepatocellular**	URI/TRIM28/MDM2/p53, SREBP1/SCD1, lactate–SREBP1/SCD1, C12ORF49–SREBP1–SCD1	URI, TRIM28, MDM2, p53, lactate, MYC, C12ORF49, SOX4, CHREBP	MUFA biosynthesis, metabolic reprogramming, TKI resistance	SCD1 inhibition (e.g., aramchol) with TKIs (donafenib) restores sensitivity; targeting SREBP1–SCD1 enhances ferroptosis	[68–73]
**Gallbladder**	circEZH2/miR-556-5p/SCD1	circEZH2, miR-556-5p, IGF2BP2	MUFA synthesis, mRNA stabilization	Targeting circEZH2/IGF2BP2/SCD1 axis restores ferroptosis and limits tumor growth	[74]
**Prostate**	LXRα/SCD1, AhR/NR4A1, EZH2/ATOH8/TCF3	TAM-secreted taurine, TCDD, NR4A1, ATOH8, EZH2	MUFA production, transcriptional repression of ferroptosis genes, epigenetic silencing of ATOH8	Targeting the EZH2/ATOH8/SCD1 axis or TAM signaling enhances ferroptosis and tumor suppression	[75, 76]
**Ovarian**	PI3K/NRF2/mTOR/SREBP1/SCD1, AMPKα/SCD1	FADS2, SGK1, SLC38A5, ATO, agrimonolide	MUFA accumulation, redox homeostasis, chemoresistance	SCD1 inhibition induces ferroptosis and apoptosis, sensitizing tumors to cisplatin, Auranofin, or olaparib	[77–82]
**Bladder**	Wnt/β-catenin/SCD1	circPKN2, LRP8	SCD1 stabilization via Wnt signaling, lipid remodeling	Targeting LRP8 or enhancing circPKN2-induced SCD1 degradation promotes ferroptosis	[83]
**Melanoma**	Wnt/β-catenin/MITF/SCD1	MITF	Membrane stabilization via MUFA synthesis	Inhibition of Wnt/β-catenin/MITF/SCD1 axis enhances ferroptosis and immunotherapy response	[84]
**Glioma**	mTOR/SCD1	lncRNA KB-1460A1.5, NADPH oxidases	Lipid redox balance maintenance	Targeting lncRNA–mTOR/SCD1 axis increases ferroptotic sensitivity	[85]
**Neuroblastoma**	PUFA/MUFA balance	PI3K/SREBP1/SCD1	MUFA enrichment	SCD1 inhibition, especially combined with PUFA delivery, restores ferroptosis and suppresses tumor growth	[86]
**AML**	PI3K/SREBP1/SCD1, ATGL/CEBPα/SCD1, circZBTB46/SCD1	PI3K, SREBP1, circZBTB46, ATGL, CEBPα, Polyphyllin I	Lipid metabolic reprogramming; MUFA synthesis; ferroptosis evasion	SCD1 or PI3K/SREBP1 inhibition induces ferroptosis and enhances therapy response	[87–89]

### Lung cancer

In lung cancer, where fluctuating oxygen tension and chronic oxidative stress are intrinsic features, tight control of membrane lipid composition is required to restrain lipid peroxidation and ferroptotic damage. In this context, SCD1 integrates environmental constraints with oncogenic and epigenetic signaling to shape lipid metabolism and ferroptosis sensitivity. The high-oxygen lung microenvironment imposes strong selective pressure for lipid remodeling programs that stabilize membranes and buffer oxidative stress, rendering SCD1-dependent MUFA synthesis a critical adaptive mechanism.

While direct evidence linking SCD1-dependent lipid desaturation to ferroptosis regulation in small cell lung cancer (SCLC) remains limited, substantial data support a central role for SCD1 in non-small cell lung cancer (NSCLC). Within non-small cell lung cancer (NSCLC), aberrant signaling pathways play a major role in sustaining SCD1- dependent ferroptosis resistance. In this setting, G-protein-coupled estrogen receptor (GPER1) has been shown to suppress ferroptosis by activating the PI3K/AKT/mTOR-SREBP1-SCD1 axis [18]. Through this pathway, GPER1 enhances SCD1 expression and lipogenesis, thereby reducing lipid ROS accumulation and ferroptotic sensitivity. Consistently, elevated expression of GPER1 and SCD1 in NSCLC specimens correlates with poor prognosis, and pharmacological inhibition of GPER1 sensitizes tumor cells to cisplatin, highlighting the functional relevance of this signaling axis.

At the epigenetic level, lymphoid-specific helicase (LSH), activated by EGLN1 and c-Myc, further reinforces ferroptosis resistance in NSCLC by transcriptionally inducing SCD1 and FADS2 [19]. This coordinated upregulation links chromatin remodeling and DNA methylation to lipid metabolic reprogramming. Beyond its canonical role in polyunsaturated fatty acid metabolism, FADS2 can alternatively desaturate saturated fatty acids such as palmitic acid to sapienic acid, a monounsaturated fatty acid that stabilizes membrane phospholipids to limit lipid peroxidation [20, 21]. Through this mechanism, FADS2 cooperates with SCD1-dependent lipid remodeling to reinforce ferroptosis resistance, particularly under hypoxic and iron-rich conditions.

In parallel, cellular plasticity further refines SCD1-dependent ferroptosis regulation in NSCLC. The EMT transcription factor Zeb1 modulates the expression of lipogenic enzymes including SCD1, FADS2, FASN, ELOVL5, and ACSL4, altering the balance between pro-ferroptotic polyunsaturated fatty acids and protective MUFAs [22]. This lipid rewiring links EMT programs to ferroptosis resistance, as pharmacological inhibition of SCD1 and FADS2 preferentially sensitizes high-Zeb1-expressing NSCLC cells to ferroptosis, providing a potential therapeutic strategy for aggressive tumor subtypes.

Consistent with this metabolic dependency, therapeutic targeting of SCD1 in NSCLC has demonstrated promising anti-tumor activity. Eupalinolide A (EA) inhibits NSCLC cell proliferation and migration by downregulating SCD1 via the AMPK/mTOR pathway, thereby inducing apoptosis and ferroptosis in vitro and suppressing tumor growth in vivo. Similarly, synthetic oxabicyclo[3.3.1]nonane derivatives show potent activity against osimertinib-resistant NSCLC, acting through SCD1 inhibition and ferroptosis induction [23].

Within this framework, lung adenocarcinoma (LUAD) further exploits SCD1-driven lipid reprogramming, but through distinct genetic determinants that shape therapy resistance and ferroptotic vulnerability. Concurrent mutations in STK11 and KEAP1, which occur frequently in KRAS-mutant LUAD, confer primary resistance to therapy and correlate with poor prognosis [24, 25]. This unfavorable clinical outcome is at least in part explained by enhanced resistance to ferroptosis, driven by the activation of lipid-protective programs centered on SCD1. Mechanistically, these co-mutations lead to the upregulation of ferroptosis-protective genes, including SCD1 and SLC7A11, through activation of the AKT/GSK3β/NRF2 axis. Increased SCD1 expression promotes MUFA synthesis, thereby buffering membrane lipids against lipid peroxidation and ferroptotic stress. CRISPR-based genetic screens and pharmacological studies validated SCD1 as a selective metabolic dependency in STK11/KEAP1 co-mutant LUAD models, in which its inhibition not only primes cancer cells for ferroptosis but also enhances the efficacy of classical ferroptosis inducers such as erastin and RSL3. Functional studies further demonstrated that loss of STK11 alone is sufficient to repress ferroptosis by increasing MUFA synthesis via SCD1, as evidenced by elevated lipid droplet accumulation, reduced lipid ROS levels, and enhanced ferroptosis resistance in mutant cells [26]. Conversely, SCD1 inhibition restores oxidative stress and ferroptotic sensitivity, highlighting its therapeutic relevance in STK11-mutant LUAD contexts.

Importantly, our group has recently provided additional evidence that lipid metabolism reprogramming is further reinforced under cancer stem-like conditions. By comparing three-dimensional vs two-dimensional cultures of primary LUAD cell lines isolated from pleural effusions, we observed a significant upregulation of SCD1 and FADS2, while ACSL4 expression remained largely unchanged between the two growth conditions [27]. These findings indicate that SCD1- and FADS2-driven lipid desaturation supports the maintenance of cancer stem cell (CSC)-like features by protecting membrane lipids from peroxidation and limiting ferroptotic vulnerability.

Consistent with the central role of SCD1 in ferroptosis protection, menin-MLL inhibitors such as MI-463 have been shown to induce ferroptosis across several cancer types, including lung cancer, through a mechanism involving SCD1 inhibition [28]. This effect is reversed by oleic acid (OA) supplementation or ferroptosis inhibitors, supporting a direct role for SCD1-dependent lipid desaturation in reinforcing lipid desaturation-dependent ferroptosis resistance.

Beyond tumor cell-intrinsic metabolism, SCD1 also contributes to ferroptosis regulation at the level of the tumor-microenvironment. In lung-to-brain metastases, lung cancer cells activate STAT3 signaling in microglia, leading to increased SCD1 expression and lipid droplet accumulation in these resident immune cells. Elevated microglia SCD1 activity dampens inflammatory response and supports a pro-tumorigenic environment that supports metastatic growth. Pharmacological inhibition of SCD1, especially in combination with blockade of the colony-stimulating factor 1 receptor (CSF1R), a key regulator of microglial and macrophage proliferation and polarization, markedly reduced brain metastatic burden in vivo [29].

Collectively, these findings indicate that lung cancer, particularly NSCLC, converges on an SCD1-dependent ferroptosis-resistant state through the integration of oncogenic, epigenetic, and microenvironmental cues. In this setting, LUAD is distinguished by the prominent role of genetic determinants, as recurrent STK11/KEAP1 co-mutations, together with EMT-associated plasticity and high oxygen exposure, reinforce a dependency on SCD1-driven MUFA synthesis to buffer membrane lipid peroxidation and sustain ferroptosis resistance across both differentiated and stem-like tumor states.

### Breast cancer

Breast cancer (BC) provides a clear example of how a tissue-specific metabolic environment amplifies SCD1-dependent ferroptosis resistance. The adipose-rich mammary niche supplies abundant lipid substrates that breast cancer cells exploit to sustain SCD1 activity, to reshape phospholipid saturation profiles by shifting the PUFA/MUFA balance away from ferroptotic vulnerability.

Within this lipid-enriched microenvironment, SCD1 acts as a central metabolic hub integrating extracellular lipid availability with oncogenic signaling pathways. Adipocyte-derived fatty acids are efficiently internalized and trafficked in breast cancer cells through fatty acid–binding protein 4 (FABP4), which indirectly supports SCD1-mediated MUFA synthesis. By channeling incoming lipids toward desaturation, FABP4 reinforces SCD1-driven lipid remodeling and ferroptosis resistance. Consistently, the upregulation of both SCD1 and FABP4 in recurrent tumors is associated with worse prognosis, and inhibiting these enzymes restores ferroptosis sensitivity, indicating their potential as therapeutic targets [30].

In addition, recent insights into acyl starvation further underscore the therapeutic relevance of lipid metabolism in ferroptosis regulation. As reviewed by Bobinski et al., limiting the availability of exogenous fatty acids not only disrupts energy homeostasis but also sensitizes tumor cells to ferroptosis by depleting their PUFA reservoirs and intersecting with immune-mediated ferroptosis [31]. In this context, sustained SCD1 activity may represent a key compensatory adaptation that allows breast cancer cells to buffer lipid stress.

At the signaling level, SCD1 is a key downstream effector of the PI3K/AKT/mTOR pathway, with SREBP1 driving SCD1 transcription to promote MUFA biosynthesis. Suppressing either SREBP1 or SCD1 restores ferroptosis sensitivity in breast cancer cells harboring PI3K mutations or PTEN loss [32]. This regulatory axis is particularly relevant in triple-negative breast cancer (TNBC), where compounds such as Formononetin and Salidroside inhibit this pathway, sensitizing TNBC cells to ferroptosis [33, 34].

Additional modulators further regulate SCD1-dependent ferroptosis resistance in TNBC. Cannabinoid receptor 1 (CB1) has been identified as an upstream regulator of SCD1 expression in TNBC, with CB1 signaling sustaining lipid desaturation and ferroptosis resistance, whereas CB1 inhibition lowers SCD1 levels and increases ferroptotic vulnerability [35]. In parallel, alternative desaturation pathways can cooperate with SCD1 to fine-tune lipid composition in breast cancer cells. Fatty acid desaturases FADS1 and FADS2 have been implicated as complementary modulators of lipid desaturation, acting alongside SCD1 to maintain a favorable balance between saturated, monounsaturated, and polyunsaturated fatty acids in TNBC [36]. Rather than functioning independently, FADS2 activity appears to support SCD1-driven protection against lipid peroxidation, thereby contributing to a multilayered desaturation network that suppresses ferroptotic vulnerability. Accordingly, co-targeting SCD1, FADS1/2, or upstream lipid transporters may offer a promising strategy to overcome this resistance.

Finally, post-transcriptional regulation adds an additional layer of control over SCD1 expression and stability. The circular RNA circSCD1 promotes ferroptosis resistance in breast cancer by preventing SCD1 degradation through binding to OTUB1, a deubiquitinating enzyme that removes ubiquitin chains from target proteins and protects them from proteasomal degradation [37]. Additionally, ADAR1/2, through their RNA editing activity, sustain SCD1 expression and protect breast cancer cells from ferroptosis, representing another mechanism of post-transcriptional regulation [38].

Recent evidence also links DNA damage response pathways to SCD1-dependent ferroptosis regulation in TNBC. Polynucleotide kinase/phosphatase (PNKP), overexpressed in TNBC and associated with poor prognosis, limits ferroptotic sensitivity by sustaining redox homeostasis and maintaining SCD1 and GPX4 expression [39]. PNKP inhibition activates STING-dependent autophagy and STAT3 suppression, promoting ferritinophagy, iron accumulation, and SCD1 downregulation, thereby enhancing ferroptosis and synergizing with doxorubicin.

Together, these observations highlight breast cancer as a model in which ferroptosis resistance is tightly coupled to the lipid-rich mammary niche. Here, the continuous influx of adipocyte-derived fatty acids, combined with layered transcriptional, post-transcriptional, and metabolic control, channels lipid utilization toward SCD1-dependent desaturation, thereby reshaping membrane composition and limiting lipid peroxidation.

### Colorectal cancer

In colorectal cancer (CRC), chronic metabolic and redox stress impose a strong selective pressure for lipid remodeling mechanisms that suppress ferroptotic vulnerability. Within this context, SCD1 emerges as a central metabolic effector that integrates redox control, oncogenic signaling, and post-transcriptional regulation to maintain a ferroptosis-resistant lipid landscape.

One of the key upstream regulators of SCD1 in CRC is TIGAR (TP53-induced glycolysis and apoptosis regulator), which is involved in controlling oxidative stress by regulating glutathione levels [40]. TIGAR is frequently overexpressed in CRC and contributes to ferroptosis resistance by sustaining antioxidant defenses. Notably, TIGAR depletion reduces SCD1 expression, leading to increased lipid peroxidation, membrane destabilization, and enhanced ferroptotic cell death, indicating that TIGAR-dependent redox buffering functionally converges on SCD1 as a downstream effector to protect CRC cells.

Beyond redox regulation, developmental and transcriptional signaling pathways further converge on SCD1 to reinforce ferroptosis resistance in CRC. Nodal, a morphogen associated with aggressive CRC phenotypes, activates the Smad2/3-SCD1 axis, thereby enhancing resistance to ferroptotic cell death [41]. In parallel, post-transcriptional mechanisms contribute to sustained SCD1 expression. Circular RNA circRHBDD1 [42] and the long noncoding RNA LINC01606 [43] stabilize SCD1 mRNA, further supporting lipid desaturation and protecting CRC cells from ferroptosis.

Pharmacological and signaling-based interventions further highlight the central role of SCD1 as a therapeutic vulnerability in CRC. SREBP1 directly promotes SCD1 transcription in CRC, sustaining MUFA production and limiting lipid peroxidation [44]. The same study demonstrated that aspirin sensitizes PIK3CA-mutant CRC cells to ferroptosis by suppressing the PI3K/AKT/mTOR/SREBP1/SCD1 signaling cascade, thereby functionally linking SREBP1-driven transcriptional control of SCD1 to therapeutic modulation of ferroptotic sensitivity.

Additionally, GPR37, a G-protein-coupled receptor upregulated in CRC, protects against ferroptosis by activating the p38/SCD1 axis [45]. Consistently, inhibition of PPP2CA, a catalytic subunit of protein phosphatase 2 A, sensitizes CRC cells to erastin-induced ferroptosis via AMPK-dependent suppression of SCD1 [46].

More recently, amino acid transport has emerged as an additional layer of ferroptosis regulation converging on SCD1 in CRC. The glutamine transporter SLC38A5 promotes ferroptosis resistance by activating the Hippo-YAP signaling pathway, leading to YAP nuclear translocation and transcriptional activation of Nrf2 [47]. This axis sustains antioxidant defenses and modulates the expression of key ferroptosis regulators, including GPX4, SLC7A11, ACSL4, and SCD1. Notably, SLC38A5 depletion sensitizes CRC cells to RSL3-induced ferroptosis, underscoring how nutrient transport, redox control, and lipid desaturation cooperate to suppress ferroptotic vulnerability.

In addition, RNA helicase DHX33 has been identified as a critical regulator of lipid metabolism in CRC, promoting the expression of FADS1, FADS2, and SCD1 [48]. Pharmacological inhibition of DHX33 with small molecules such as KY386 induces ferroptosis selectively in cancer cells, highlighting DHX33 as a promising therapeutic target that converges on SCD1-dependent ferroptotic vulnerability.

In parallel, resistance to targeted therapies in CRC is reinforced by lipid metabolic reprogramming driven by MYLK4 [49]. MYLK4 promotes ferroptosis resistance by inducing MDM2-dependent p53 degradation, thereby relieving p53-mediated repression of SCD1. Elevated MYLK4 expression correlates with increased SCD1 levels, regorafenib resistance, and poor prognosis in CRC patients. Importantly, combined inhibition of SCD1 and regorafenib restores ferroptotic sensitivity in organoid and xenograft models derived from p53-wild-type CRC, highlighting the MYLK4-p53-SCD1 axis as a clinically actionable vulnerability.

Collectively, these findings indicate that colorectal cancer integrates chronic redox stress with layered genetic, transcriptional, and post-transcriptional regulatory networks to establish SCD1 as a central determinant of ferroptosis resistance. In this context, sustained oxidative pressure, together with oncogenic and RNA-mediated stabilization mechanisms, converges on SCD1-driven lipid desaturation as a common adaptive strategy that preserves membrane integrity and limits ferroptotic cell death.

### Pancreatic cancer

Pancreatic cancer (PC), characterized by profound metabolic rewiring and intrinsic therapy resistance, exploits SCD1 as a central node linking oncogenic signaling to ferroptosis suppression. In this context, SCD1 functions both as a downstream effector and as an active regulator within multiple oncogenic pathways that sustain lipid desaturation and protect tumor cells from oxidative damage. One key regulatory pathway involves the tumor suppressor FBW7, which is frequently inactivated by the MEK/ERK signaling. Loss of FBW7 relieves NR4A1-mediated repression of SCD1, thereby sustaining MUFA synthesis and ferroptosis resistance [50]. Conversely, restoration of FBW7 expression or direct pharmacological inhibition of SCD1 enhances lipid peroxidation, sensitizes PC cells to ferroptosis and apoptosis, and potentiates the efficacy of gemcitabine, supporting a combinatorial therapeutic strategy.

Beyond FBW7, transcriptional control of lipid metabolism represents a major mechanism sustaining SCD1 expression in PC. In particular, the BHLHE40/SREBP1/SCD1 axis acts as a critical transcriptional cascade linking lipogenesis to ferroptosis resistance [51]. BHLHE40 induces SREBP1 transcription, thereby activating SCD1 and promoting MUFA biosynthesis. This MUFA-enriched state rewires phospholipid composition and constrains ferroptotic lipid oxidation. Pharmacological inhibition of this pathway, for example using the SREBP inhibitor fatostatin, suppresses tumor growth and restores ferroptotic sensitivity in pancreatic cancer models, highlighting the therapeutic relevance of targeting SCD1-driven lipid desaturation.

Consistent with this model, multiple metabolic regulators converge on SCD1 to reinforce ferroptosis resistance and chemoresistance in PC. The nuclear coactivator NCOA6 enhances SCD1 expression via SREBP1 activation, and its silencing reduces lipid desaturation, increases lipid peroxidation, and sensitizes tumor cells to both ferroptosis and gemcitabine [52]. Similarly, the glutamine transporter SLC38A5, which is upregulated in drug-resistant PC, sustains mTOR/SREBP1/SCD1 signaling. Inhibition of SLC38A5 disrupts glutamine metabolism, weakens antioxidant defenses, and promotes ferroptosis, thereby restoring chemosensitivity [53].

Additional layers of regulation further underscore the adaptability of SCD1-dependent ferroptosis resistance in pancreatic tumors. ZNF488 directly activates SCD1 transcription, promoting tumor growth and ferroptosis resistance, whereas its knockdown has the opposite effect [54].

In pancreatic neuroendocrine tumors (pNETs), SCD1 is regulated through the MEN1/mTOR axis, where MEN1 suppresses SCD1 expression, leading to PUFA accumulation and increased ferroptotic vulnerability [55]. In this setting, combining mTOR inhibitors such as everolimus with ferroptosis inducers shows promising therapeutic synergy.

Moreover, recent evidence highlights CD147 as an upstream modulator of SCD1 in pancreatic ductal adenocarcinoma [56]. The small-molecule inhibitor dracorhodin perchlorate (DP) promotes CD147 degradation, leading to downregulation of the SREBP1/SCD1 pathway and a shift in the balance between polyunsaturated and monounsaturated fatty acids. This metabolic perturbation triggers ferroptosis and potentiates the efficacy of gemcitabine in vitro and in vivo, demonstrating that targeting CD147 can effectively disrupt SCD1-mediated ferroptosis resistance and enhance therapeutic response.

Taken together, these observations show that in pancreatic cancer, ferroptosis resistance is sustained by the tight coupling of oncogenic signaling and lipid metabolic control, whereby multiple pathways converge functionally on SCD1-dependent MUFA synthesis. This integration not only limits lipid peroxidation but also supports the pronounced resistance of pancreatic tumors to chemotherapy.

### Gastric cancer

In gastric cancer (GC), dysregulated lipid metabolism is exploited to evade ferroptotic cell death. In this tumor type, SCD1 has emerged as a critical regulator of lipid metabolism, ferroptosis resistance, and cancer stem cell maintenance, contributing significantly to tumor growth, therapeutic evasion, and poor clinical outcomes. Elevated expression of SCD1 has been observed in GC tissues and cell lines, where it facilitates the biosynthesis of MUFAs such as OA. These lipids reprogram membrane saturation dynamics, reducing ferroptotic susceptibility [57]. Inhibition of SCD1 results in suppressed proliferation, reduced migration, and increased sensitivity to ferroptosis inducers, positioning the enzyme as both a prognostic biomarker and a therapeutic target in gastric malignancy.

One of the key mechanistic axes involves the SCD1/Wnt/β-catenin signaling pathway, as shown in studies on poorly cohesive carcinoma (PCC). Treatment with Jianpi Yangzheng Xiaozheng granule (JPYZXZ), a traditional Chinese herbal formulation, induces ferroptosis and disrupts lipid metabolism in GC cells by downregulating SCD1, GPX4, xCT, and β-catenin, leading to oxidative stress and impaired cellular lipid homeostasis [58]. Notably, the effects of JPYZXZ mirror those of pharmacological or genetic SCD1 inhibition, and are nullified in SCD1-silenced cells, further confirming the upstream regulatory role of SCD1 in this pathway.

These observations indicate that SCD1-dependent lipid desaturation does not operate in isolation, but instead orchestrates a broader metabolic program that links ferroptosis resistance to stemness and long-term tumor persistence.

Within this integrated framework, SCD1 promotes stemness and ferroptosis resistance in gastric cancer stem cells (GCSCs) by coupling lipid desaturation to cholesterol metabolism [59]. Mechanistically, SCD1 enhances transcription of squalene epoxidase (SQLE) by relieving p53-mediated repression, leading to increased cholesterol synthesis. This cholesterol-enriched state activates mTOR signaling, which simultaneously suppresses ferroptosis and sustains CSC self-renewal capacity, thereby functionally linking lipid metabolism, redox protection, and stemness maintenance. In vivo, both SCD1 knockdown and dietary modulation of cholesterol reduce tumor growth and restore ferroptotic sensitivity, supporting the relevance of the SCD1/SQLE/cholesterol/mTOR axis in disease progression.

This stemness-associated metabolic state is further stabilized by post-transcriptional mechanisms that ensure persistent SCD1 expression under therapeutic stress. In this context, exosomal lncRNA lncFERO, secreted by GC cells in response to chemotherapy, enhances SCD1 expression in GCSCs by binding its mRNA and recruiting hnRNPA1, thereby stabilizing the transcript and reinforcing lipid metabolic reprogramming [60]. By sustaining SCD1 levels during treatment, the exo-lncFERO/hnRNPA1/SCD1 axis locks cancer cells into a ferroptosis-resistant, stem-like state, promoting tumor persistence and acquired chemoresistance in advanced GC.

In addition, recent evidence identifies Tropomyosin 4 (TPM4) as an upstream regulator of SCD1 in gastric cancer. TPM4 expression is elevated in GC tissues and positively correlates with SCD1 levels [61]. Functional studies show that TPM4 knockdown inhibits GC cell proliferation, induces ferroptosis, and slows tumor growth in vivo by suppressing SCD1 expression, further highlighting SCD1 as a central mediator of ferroptosis resistance and tumor progression in GC.

Together, these findings highlight gastric cancer as a context in which SCD1-dependent lipid desaturation is tightly linked to the maintenance of cancer stem cell properties. Through the integration of lipid and cholesterol metabolism, SCD1 sustains a stem-like, ferroptosis-resistant state that promotes long-term tumor persistence and therapy escape.

### Esophageal cancer

In esophageal squamous cell carcinoma (ESCC), metabolic stress and nutrient availability critically modulate SCD1-dependent ferroptosis sensitivity, thereby influencing both therapeutic response and metastatic behavior. Under these conditions, SCD1 functions as a key metabolic regulator that shapes lipid composition, controls susceptibility to lipid peroxidation, and ultimately determines ferroptotic vulnerability.

Consistent with this role, pharmacological inhibition of SCD1 using the small-molecule inhibitor MF-438 enhances radiotherapy efficacy by promoting ferroptosis and immunogenic cell death, supporting SCD1 as both a radiosensitizing target and a potential prognostic biomarker in ESCC [62]. These findings indicate that SCD1-dependent lipid desaturation actively contributes to radioresistance by limiting oxidative lipid damage under therapeutic stress.

However, SCD1 activity exerts context-dependent effects, particularly in the regulation of metastatic dissemination. Reduced SCD1 expression can paradoxically favor metastasis under specific conditions. The transcription factor BACH1, which is associated with lymph node metastasis, promotes ferroptosis by repressing SCD1 expression [63]. This increase in ferroptotic sensitivity leads to the release of OA, generating a chemoattractive gradient that facilitates lymphatic spread. Importantly, supplementation with OA rescues BACH1-overexpressing cells from ferroptosis and reverses their metastatic phenotype, highlighting the BACH1-SCD1-OA axis as a critical determinant of metastasis behavior.

In addition to transcriptional regulation, microenvironmental nutrient imbalance further shapes SCD1-dependent ferroptosis resistance in ESCC. Zinc deficiency (ZD) promotes tumor cell proliferation, migration, and invasion while suppressing lipid peroxidation and ferroptotic response [64]. Mechanistically, ZD downregulates p-AMPK and activates the SREBP1/SCD1 axis, enhancing MUFA synthesis and reinforcing membrane protection against oxidative damage. This metabolic adaptation represents an escape route exploited by ESCC cells to survive under oxidative and nutrient stress.

Overall, in ESCC, SCD1 functions as a context-dependent regulator that links ferroptosis control to metastatic behavior. While SCD1 activity protects tumor cells from therapy-induced lipid peroxidation, its suppression can promote ferroptosis-associated lipid release and facilitate metastatic dissemination, highlighting a direct interplay between ferroptotic sensitivity and tumor spread.

### Anaplastic thyroid carcinoma

In anaplastic thyroid carcinoma (ATC), one of the most aggressive endocrine malignancies, rapid tumor growth is sustained by enhanced lipogenic activity that supports membrane biosynthesis and redox balance. In this context, lipid metabolic rewiring contributes to ferroptosis-associated radioresistance. YTHDF2-mediated stabilization of m6A-modified SREBF1 transcripts enhances SCD1 expression, promoting monounsaturated fatty acid biosynthesis and limiting lipid peroxidation [65]. Inhibition of YTHDF2 restores ferroptotic susceptibility and sensitizes ATC models to radiotherapy, identifying an epitranscriptomic mechanism that reinforces the SREBF1–SCD1 axis in this aggressive endocrine malignancy.

Thus, ATC exemplifies a tumor type in which epitranscriptomic control of lipid metabolism sustains SCD1-dependent ferroptosis resistance, thereby linking lipid desaturation to radioresistant tumor growth.

### Head and neck squamous cell carcinoma

In head and neck squamous cell carcinoma (HNSCC), tumor cells adapt to fluctuating nutrient availability and harsh microenvironmental conditions by reshaping lipid metabolism, which in turn influences ferroptosis sensitivity. Recent evidence indicates that ferroptosis sensitivity is tightly regulated by lipid metabolic pathways converging on SCD1. The DDR1-Akt/mTORC1-SREBP1 axis has been identified as a key driver of monounsaturated fatty acid (MUFA) biosynthesis, sustaining ferroptosis resistance [66]. Disruption of this signaling cascade enhances lipid peroxidation, promotes immunogenic ferroptotic cell death, and increases the efficacy of carbon ion radiotherapy, highlighting the therapeutic relevance of targeting SCD1-dependent metabolic programs.

In parallel, metabolic compartmentalization within the tumor microenvironment further shapes ferroptosis vulnerability. Acid-exposed cancer cells display increased dependence on fatty acid desaturation, and SCD1 inhibition selectively induces ferroptosis in these niches while limiting unsaturated fatty acid availability to neighboring hypoxic cells [67].

In conclusion, in HNSCC, ferroptosis sensitivity is shaped by the interplay between oncogenic signaling and microenvironmental metabolic compartmentalization, with SCD1-dependent lipid desaturation supporting adaptation to spatially distinct nutrient and stress conditions within the tumor.

### Hepatocellular carcinoma

In Hepatocellular Carcinoma (HCC), a highly metabolically active tumor type arising in a lipid- and nutrient-rich hepatic environment, SCD1 integrates oncogenic signaling with altered nutrient fluxes to sustain lipid homeostasis and protect cancer cells from ferroptosis. In this cellular environment, SCD1 plays a pivotal role in promoting tumor growth and conferring resistance to therapy, particularly to tyrosine kinase inhibitors (TKIs).

A key regulatory mechanism involves the oncogene URI, which interacts with TRIM28 and MDM2 to promote p53 degradation [68]. Because p53 normally represses SCD1 transcription, loss of p53 function results in SCD1 up-regulation, enhanced MUFA synthesis, and increased protection against lipid peroxidation and ferroptotic cell death. Clinically, co-expression of URI and SCD1 correlates with poor prognosis and reduced response to TKIs, particularly in p53 wild-type HCC. Importantly, combining the SCD1 inhibitor aramchol with donafenib, a multi-kinase inhibitor targeting Raf kinase and various receptor tyrosine kinases, produces synergistic anti-tumor effects in SCD1-high, p53-intact tumors, offering a strategy to overcome TKI resistance.

Beyond oncogenic signaling, metabolic intermediates further reinforce SCD1-dependent ferroptosis resistance in HCC. Lactate accumulation, a hallmark of altered hepatic metabolism, activates the SREBP1-SCD1 axis, thereby sustaining MUFA production and limiting lipid peroxidation [69]. Consistently, natural compounds such as aurantio obtusin (AO) enhance ferroptotic sensitivity by downregulating SCD1 and GPX4, underscoring the therapeutic potential of targeting lipid metabolic adaptations in HCC [70].

Further upstream regulators of SCD1 have been identified in HCC. C12ORF49, a newly characterized modulator of SREBP1, is overexpressed in HCC tissues and promotes MUFA synthesis through SREBP1/SCD1 signaling, thereby inhibiting ferroptosis [71]. Knockdown of C12ORF49 induces ferroptosis and suppresses tumor growth, while combined targeting of C12ORF49 and Sorafenib shows synergistic anti-tumor effects, highlighting C12ORF49 as a potential therapeutic target to enhance the efficacy of standard treatments.

Similarly, the transcription factor SOX4 contributes to ferroptosis resistance by reprogramming fatty acid metabolism via the CHREBP/SCD1 pathway [72]. In HCC, SOX4 upregulation promotes MUFA production, suppresses lipid peroxidation, and concurrently enhances angiogenesis and tumor progression. These findings underscore how transcriptional regulators can converge on SCD1 to protect HCC cells from ferroptosis while supporting malignant phenotypes.

At the molecular subtype level, recent pan-cohort analyses of more than 3900 HCC samples have identified two ferroptosis-associated subtypes: C1, characterized by low metabolism and high immune infiltration, and C2, defined by high metabolic activity and reduced immune engagement [73]. Notably, SCD1 methylation is enriched in the poor-prognosis C1 subtype, which also exhibits frequent BAP1 mutations and MYC amplification. These features link epigenetic regulation of SCD1 to ferroptosis-related tumor heterogeneity and immune contexture, positioning SCD1 not only as a metabolic effector but also as a biomarker with potential relevance for patient stratification and immunotherapy.

Overall, in hepatocellular carcinoma, SCD1-dependent lipid desaturation operates at the intersection of nutrient-rich metabolic conditions and oncogenic signaling, where its activation not only limits ferroptotic damage but also shapes therapeutic response and tumor heterogeneity.

### Gallbladder cancer

Within the spectrum of gastrointestinal malignancies, gallbladder cancer (GBC) further illustrates how post-transcriptional regulation converges on SCD1 to promote ferroptosis resistance. In GBC, SCD1 supports tumor progression by sustaining lipid metabolic reprogramming and limiting lipid peroxidation. A key regulatory mechanism involves the circular RNA circEZH2, which enhances SCD1 expression by sponging miR-556-5p, thereby increasing MUFA synthesis and suppressing ferroptotic cell death [74]. This effect is further amplified by IGF2BP2, an RNA-binding protein that stabilizes circEZH2 and prolongs its regulatory activity. Together, the circEZH2/IGF2BP2/SCD1 axis establishes a post-transcriptional feed-forward loop that promotes lipid desaturation and ferroptosis resistance, highlighting SCD1 as a downstream effector of noncoding RNA-mediated metabolic control in gallbladder cancer.

### Prostate cancer

Prostate cancer (PCa) exhibits a marked dependence on lipid metabolism and develops within a tumor microenvironment strongly influenced by immune-derived signals and environmental cues, which together create a biologically relevant context for SCD1-mediated ferroptosis resistance.

Within this lipid-dependent setting, SCD1 acts as a central integrator of extrinsic cues that modulate ferroptotic sensitivity. Tumor-associated macrophages (TAMs) contribute to this process by secreting taurine, which activates the LXRα/SCD1 axis, thereby enhancing MUFA production and protecting tumor cells from lipid peroxidation and ferroptotic cell death [75]. In contrast, exposure to environmental toxins such as 2,3,7,8-tetrachlorodibenzo-p-dioxin (TCDD) suppress SCD1 expression via the AhR/NR4A1 pathway, increasing ferroptotic vulnerability. Importantly, NR4A1 overexpression counteracts this effect by restoring SCD1 levels, highlighting the dynamic and reversible regulation of SCD1 in response to environmental stressors.

Beyond microenvironmental regulation, cell-intrinsic transcriptional and epigenetic mechanisms further fine-tune SCD1 expression in prostate cancer. The basic helix-loop-helix (bHLH) transcription factor ATOH8 has emerged as a key determinant of ferroptosis sensitivity through direct repression of SCD1 transcription.

ATOH8 overexpression enhances ferroptosis susceptibility, whereas its loss promotes ferroptosis evasion. At the molecular level, ATOH8 forms a transcriptional repressive complex with another bHLH factor, TCF3, to suppress SCD1 expression. This axis is itself epigenetically regulated, as EZH2-mediated H3K27me3 deposition silences ATOH8, thereby relieving repression of SCD1 and promoting ferroptosis resistance. Consistently, pharmacological inhibition of EZH2 synergizes with ferroptosis inducers to suppress tumor growth both in vitro and in vivo, underscoring the therapeutic relevance of the EZH2/ATOH8/SCD axis [76].

In prostate cancer, SCD1-dependent ferroptosis resistance emerges from the dynamic integration of immune-derived signals, environmental exposures, and epigenetic-transcriptional control, allowing tumor cells to flexibly modulate lipid desaturation and adapt to changing microenvironmental conditions.

### Ovarian cancer

In ovarian cancer (OC), particularly in peritoneal metastases and malignant ascites, a lipid-rich microenvironment favors SCD1-dependent metabolic adaptations that couple chemoresistance with ferroptosis evasion. In this setting, elevated SCD1 expression cooperates with FADS2 to promote the accumulation of monounsaturated and unsaturated fatty acids, reshaping phospholipid composition and buffering ferroptotic stress. Accordingly, simultaneous inhibition of SCD1 and FADS2 disrupts mitochondrial redox balance and induces ferroptosis, an effect that is further enhanced by cisplatin treatment, highlighting lipid desaturation as a metabolic liability that can be therapeutically exploited [77].

Beyond ferroptosis alone, SCD1 inhibition activates a broader cell death program in OC. By reducing phospholipid content and increasing pro-apoptotic ceramides, SCD1 blockade triggers both ferroptosis and apoptosis, thereby engaging dual cytotoxic pathways that may limit adaptive resistance [78]. Consistent with this concept, SCD1 inhibition sensitizes ovarian tumors to ferroptosis inducers such as RSL3 and Auranofin, resulting in marked tumor regression in preclinical models.

These observations have spurred the development of multiple strategies to therapeutically target SCD1. Natural compounds such as agrimonolide, an isocoumarin isolated from Agrimonia Pilosa, suppress SCD1 expression, elevate intracellular ROS and iron levels, and impair redox homeostasis, thereby promoting ferroptosis in OC cells [79].

In parallel, hypoxia-responsive polymeric micelles co-delivering an SCD1 inhibitor and a ferroptosis inducer have demonstrated potent antitumor activity by selectively suppressing SCD1 in hypoxic tumor regions, where ferroptosis resistance is most pronounced [80].

At the signaling level, SCD1-dependent ferroptosis resistance is further reinforced by oncogenic stress pathways. In high-grade serous OC (HGSOC), SGK1, a downstream effector of the PI3K pathway, promotes ferroptosis evasion through NRF2 activation and sustained mTOR/SREBP1/SCD1 signaling [81]. Disruption of the SGK1/SCD1 axis increases iron accumulation and lipid peroxidation, re-sensitizing tumors to ferroptotic cell death.

Finally, combinatorial therapeutic approaches underscore the centrality of SCD1 in therapy resistance. The combination of arsenic trioxide (ATO) with the PARP inhibitor olaparib effectively induces ferroptosis in platinum-resistant ovarian cancer by activating AMPKα and suppressing SCD1, thereby amplifying both apoptotic and ferroptotic cytotoxicity [82].

In ovarian cancer, SCD1-dependent lipid desaturation supports adaptation to lipid-rich peritoneal environments while enabling the integration of ferroptosis and apoptosis resistance, thereby contributing to the development of multifaceted therapy resistance.

### Bladder cancer

In bladder cancer (BC), ferroptosis sensitivity is modulated by opposing regulatory circuits that converge on SCD1, underscoring the context-dependent role of lipid desaturation in tumor survival. Rather than acting as a universal pro-survival factor, SCD1 sits at the center of antagonistic signaling pathways that differentially tune ferroptotic vulnerability.

On one hand, the circular RNA circPKN2 promotes ferroptosis by facilitating SCD1 degradation and concomitantly suppressing Wnt/β-catenin signaling thereby enhancing lipid peroxidation and ferroptotic cell death. On the other hand, LRP8 exerts the opposite effect by activating the Wnt/β-catenin/SCD1 axis, reinforcing MUFA synthesis and protecting BC cells from ferroptosis. This dual regulatory architecture positions SCD1 as a molecular switch that integrates antagonistic signals to determine ferroptotic vulnerability in bladder cancer, indicating that selectively disrupting pro-survival Wnt/β-catenin signaling or enhancing SCD1 degradation may restore ferroptosis sensitivity [83].

### Melanoma

In melanoma, signaling pathways that govern differentiation and immune evasion intersect with SCD1-dependent lipid remodeling to modulate ferroptosis sensitivity. Wnt/β-catenin signaling, a key driver of melanoma differentiation and immune exclusion, promotes ferroptosis resistance by sustaining SCD1 expression. Inhibition of this pathway increases lipid peroxidation and enhances ferroptotic vulnerability. Acting downstream of the melanocyte lineage transcription factor MITF, SCD1 contributes to ferroptosis evasion by maintaining a lineage-coupled lipid desaturation program that constrains ferroptosis. Through this Wnt/β-catenin/MITF/SCD1 axis, melanoma cells couple lineage identity with metabolic protection from ferroptosis. Consequently, disrupting this axis may not only restore ferroptosis sensitivity but also improve the efficacy of immunotherapies, including anti-PD-1 treatment, by weakening metabolic barriers to immune-mediated tumor clearance [84]. Through this Wnt/β-catenin/MITF/SCD1 axis, melanoma cells couple lineage identity with metabolic protection from ferroptosis.

### Glioma

In gliomas, ferroptosis resistance arises from the interplay between lipid metabolism, redox regulation, and non-coding RNA-mediated control of SCD1. In particular, SCD1 regulation involves crosstalk with long non-coding RNAs, such as KB-1460A1.5, which modulates the mTOR/SCD1 signaling axis [85]. Through this pathway, lncRNA-driven signaling sustains SCD1 expression and lipid desaturation, thereby limiting lipid peroxidation and buffering oxidative stress.

Disruption of this regulatory network markedly alters cellular redox balance and ferroptosis sensitivity, underscoring the functional relevance of SCD1 as a downstream effector of non-coding RNA-dependent metabolic reprogramming. In addition, the interaction between SCD1 activity and NADPH oxidases in glioma cells reveals an additional layer of redox control, in which lipid desaturation and ROS production are tightly coordinated to restrain ferroptotic damage.

In gliomas, SCD1-dependent lipid desaturation is tightly coordinated with non-coding RNA signaling and redox control, enabling tumor cells to fine-tune oxidative stress and limit ferroptotic damage.

### Neuroblastoma

Beyond gliomas, recent evidence extends the relevance of SCD1-dependent ferroptosis control to neural crest-derived tumors. Neuroblastoma (NB) has recently emerged as a ferroptosis-susceptible pediatric malignancy, albeit with marked heterogeneity across high-risk models. Metabolic screening identified SCD1 as a key ferroptosis resistance factor, with genetic or pharmacological inhibition restoring ferroptotic sensitivity in resistant NB cells and correlating with poor patient prognosis [86]. Mechanistically, SCD1 inhibition increased the PUFA/MUFA ratio, enhancing lipid peroxidation and ferroptotic cell death, which was further potentiated by arachidonic acid supplementation. Notably, nanoparticle-mediated PUFA delivery synergized with SCD1 blockade to suppress tumor growth in vivo without systemic toxicity.

In neuroblastoma, SCD1-dependent lipid remodeling acts as a critical safeguard against ferroptosis, with its inhibition exposing a therapeutically exploitable vulnerability that can be further amplified by PUFA-based strategies.

### Acute myeloid leukemia

In acute myeloid leukemia (AML), lipid metabolic reprogramming constitutes a key adaptive mechanism enabling ferroptosis evasion. SCD1 contributes to ferroptosis resistance by reshaping lipid composition and preserving a desaturation-driven ferroptosis-evading phenotype. At the post-transcriptional level, the circular RNA circZBTB46, which is upregulated in AML, promotes ferroptosis resistance by increasing SCD1 expression; conversely, inhibition of circZBTB46 reduces SCD1 levels and sensitizes AML cells to ferroptosis [87]. These findings highlight a non-coding RNA-dependent layer of SCD1 regulation that sustains lipid metabolic adaptations in leukemic cells.

More recently, lipid catabolism has emerged as an additional upstream regulator of SCD1-dependent ferroptosis resistance. Adipose triglyceride lipase (ATGL) is aberrantly upregulated in AML, particularly in FLT3-ITD-mutated disease, where it suppresses ferroptosis and promotes leukemic progression by activating the CEBPα/SCD1 axis. ATGL-driven SCD1 upregulation confers resistance to ferroptosis and contributes to reduced sensitivity to the FLT3 inhibitor gilteritinib, while pharmacological inhibition of ATGL restores ferroptotic vulnerability and enhances therapeutic efficacy in vitro and in vivo [88].

Consistently, pharmacological targeting of this axis can restore ferroptotic vulnerability. Polyphyllin I (PPI) has been shown to induce ferroptosis by suppressing the PI3K/SREBP1/SCD1 signaling pathway, leading to impaired lipid desaturation and enhanced oxidative damage [89].

Overall, in AML, SCD1 functions as a metabolic checkpoint that integrates lipid synthesis, lipolysis-derived signaling, and oncogenic inputs to sustain ferroptosis resistance, revealing a targetable vulnerability in leukemic cells.

## Comparative synthesis of SCD1-driven ferroptosis resistance across tumor types

In the previous section we have reviewed a wealth of evidence accumulated over the last years showing that across distinct cancer types, SCD1 is a conserved ferroptosis suppressor whose regulation and functional relevance are profoundly shaped by tissue-specific metabolic constraints and microenvironmental pressures. Although SCD1 activity is thought to contribute to MUFA-mediated membrane stabilization and reduced lipid peroxidation, the upstream regulatory pathways and their tumor-specific contexts show considerable variability. In highly oxidative environments such as the lung, elevated oxygen tension and chronic redox stress favor SCD1 upregulation downstream of NRF2- and oncogene-driven pathways, enabling protection against lipid peroxidation. In contrast, adipose-rich niches such as the breast and peritoneal cavity support SCD1 activity through abundant lipid availability and enhanced fatty acid trafficking, coupling extracellular lipid uptake with endogenous desaturation programs. Metabolically demanding organs, including the liver and pancreas, further amplify SCD1 dependency through nutrient-sensing and lipogenic transcriptional networks centered on SREBP1, allowing tumor cells to buffer ferroptotic stress under conditions of high metabolic flux and therapeutic pressure.

Additional layers of regulation contribute to this heterogeneity. In several tumor types, including colorectal, gastric, ovarian, and prostate cancers, SCD1 expression is reinforced by non-coding RNA-mediated stabilization, epigenetic remodeling, or immune-derived signals, highlighting how ferroptosis resistance is progressively consolidated through multi-level regulatory circuits. Conversely, tumors such as bladder cancer and melanoma illustrate how opposing signaling inputs dynamically tune SCD1 activity, positioning lipid desaturation at the interface between ferroptosis sensitivity, metastatic behavior, and immune response.

Other malignancies, including neuroblastoma, anaplastic thyroid carcinoma, and head and neck squamous cell carcinoma, similarly illustrate how diverse oncogenic and microenvironmental contexts converge on SCD1 to modulate ferroptosis vulnerability.

**Fig. 4:**
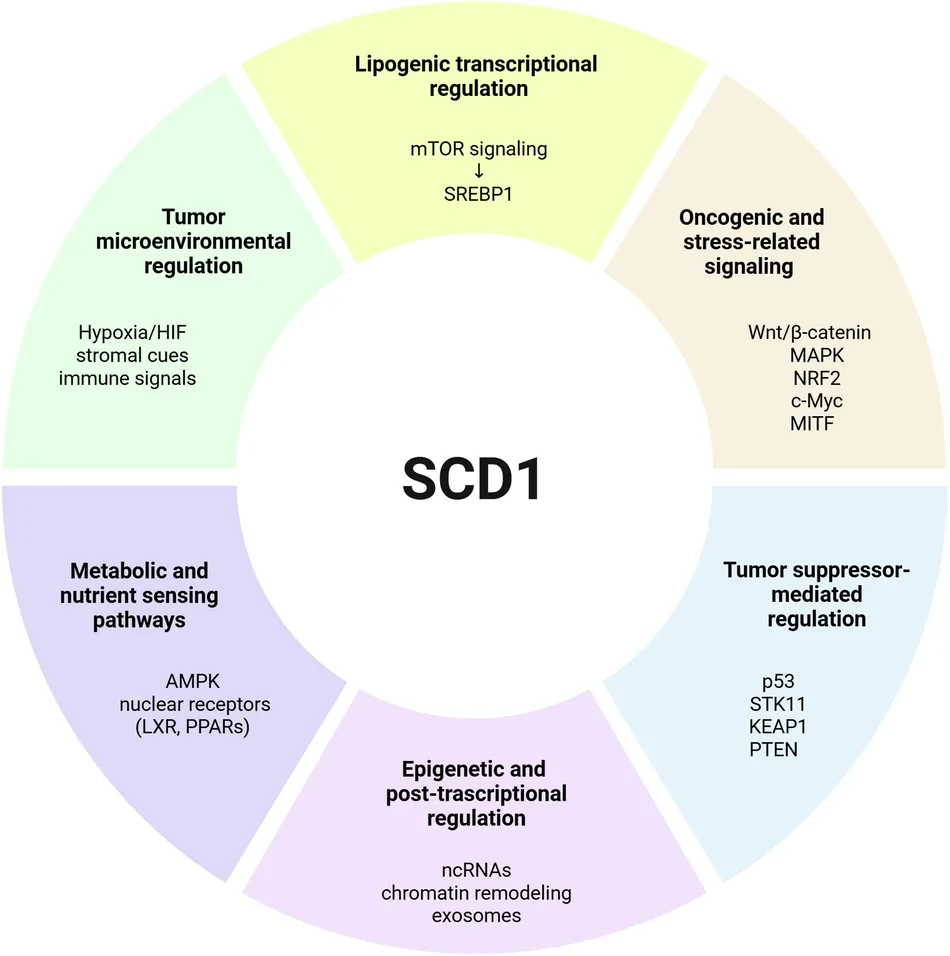
Overview of the main regulatory mechanisms converging on SCD1 in cancer. Created in BioRender. Ascenzi, F. (2026) https://BioRender.com/b6el3rw.

Notably, despite these context-dependent differences, the recurrent convergence on SCD1 underscores a unifying principle: diverse oncogenic, metabolic, and microenvironmental signals are selectively wired to preserve lipid desaturation as a dominant strategy to evade ferroptotic cell death (see Fig. 4 for an overview of the main regulatory mechanisms). This convergence provides the mechanistic rationale for comparing these tumor types, explaining why SCD1 represents a shared vulnerability across heterogeneous malignancies, while also emphasizing that effective therapeutic targeting must account for tissue-specific regulatory landscapes and adaptive metabolic states. These considerations provide a conceptual framework for better shaping therapeutic strategies targeting the SCD1-ferroptosis axis as discussed below.

## Targeting the SCD1-ferroptosis axis: a novel therapeutic strategy

Recent advances in the mechanistic understanding of SCD1-mediated ferroptosis resistance have paved the way for therapeutic strategies that so far been tested exclusively in preclinical cancer models. Prototypical synthetic inhibitors such as A939572, MF-438, and CAY10566 directly block SCD1 enzymatic activity, resulting in increased lipid peroxidation and heightened vulnerability to ferroptosis. In parallel, several natural compounds (e.g., agrimonolide) and metabolic modulators (e.g., PI3K/AKT-mTOR-SREBP1) have been reported to indirectly suppress SCD1 expression or function through upstream signaling pathways, although their specificity and translational potential remain less well defined.

Small-molecule SCD1 inhibitors consistently trigger ferroptotic cell death across multiple preclinical cancer models and exhibit strong synergy when combined with ferroptosis inducers such as erastin or RSL3 [20, 28, 75]. Table 2 summarizes representative preclinical studies supporting these combinatorial effects. In addition, SCD1 inhibition has been reported to enhance the efficacy of radiotherapy and platinum-based chemotherapy [62, 90, 91].

**Table 2:** Preclinical statistical evidence for combinations of SCD1 small-molecule inhibitors and ferroptosis inducers.

Cancer model	SCD1 small-molecule inhibitor	Ferroptosis inducer	Experimental system	Evidence of enhanced ferroptosis	Statistical support	Reference
Breast and lung cancer models (Zeb1-high mesenchymal context; MDA-MB-231, A549, H358)	MF-438; CAY10566	ML210; RSL3	In vitro viability, lipid peroxidation, ex vivo tumor slices	Pharmacological SCD1 inhibition significantly potentiates GPX4 inhibitor–induced ferroptotic cell death; effect rescued by ferrostatin-1 and oleic acid	Two-way ANOVA and multiple comparisons tests, P < 0.0001	[22]
Ovarian cancer (ascites-derived OVCA433 cells)	CAY10566	RSL3; erastin	In vitro co-culture system	SCD1 blockade enhances ferroptotic cell death and lipid peroxidation in combination treatments	Student’s t-test / ANOVA, P < 0.05	[77]
Multiple tumor recurrence models	Ab142089; DC5893	Erastin; RSL3	In vitro and in vivo models	Combined lipid desaturation blockade increases ferroptosis sensitivity and reduces tumor recurrence	Statistical significance reported in original analyses (P < 0.05)	[30]

Combining SCD1 blockade with immunotherapies is emerging as another promising approach, as ferroptosis can enhance antitumor immunity by promoting immunogenic cell death, improving T-cell recruitment, and repolarizing macrophages towards pro-inflammatory phenotypes [92]. Nevertheless, given the central role of lipid metabolism in immune function, the effects of SCD1 inhibition on immune cells must be carefully evaluated. Prolonged blockade could inadvertently impair antitumor T-cell activation, persistence, or effector function by altering lipid composition within the tumor microenvironment, highlighting the importance of selectively targeting tumor-intrinsic SCD1 activity while preserving immune competence.

Although no direct SCD1 inhibitors have yet advanced to clinical trials in oncology, several ferroptosis-inducing agents are currently under clinical evaluation. These include sorafenib and APR-246 (eprenetapopt), which exhibit ferroptotic activity and are being tested in combination regimens across different cancer types [90, 91, 93–95]. These studies provide an important clinical framework for integrating ferroptosis-based strategies into cancer therapy.

By contrast, SCD1 inhibitors have already entered the clinic in metabolic diseases. The partial SCD1 inhibitor Aramchol demonstrated reductions in hepatic fat content in patients with non-alcoholic fatty liver disease and non-alcoholic steatohepatitis, though systemic safety concerns were noted [96, 97]. Similarly, clinical trials of SCD1 inhibitors in neurological disorders were initiated but ultimately discontinued due to on-target toxicities [98], underscoring that chronic systemic SCD1 inhibition can be limited by adverse effects in skin, liver, and other tissues, narrowing the therapeutic window [99].

To overcome these challenges, two complementary directions are being actively explored. First, advanced drug delivery technologies, including liposomal or polymeric nanoparticles and biomimetic carriers, aim to enhance tumor targeting, improve pharmacokinetics, and limit off-tumor exposure of SCD1 inhibitors. In line with this concept, lipid-based nanodelivery systems have been used to modulate ferroptosis sensitivity, as shown in high-risk neuroblastoma, where PUFA-loaded nanoparticles synergize with SCD1 inhibition to enhance lipid peroxidation and ferroptotic tumor cell death in vivo [86].

Second, tumor-activated prodrugs are designed to remain inert in circulation and become activated only in response to hypoxia, acidic pH, or tumor-specific enzymatic activity, thereby restricting SCD1 inhibition to the malignant compartment [100–105].

In this context, intermittent or adaptive dosing schedules, in which SCD1 inhibition is applied in short pulses rather than continuously, may further mitigate cumulative toxicity while preserving the ability to transiently lower the ferroptosis threshold prior to chemotherapy, radiotherapy, or ferroptosis induction [32, 99]. Collectively, these observations suggest that the therapeutic potential of SCD1 targeting will most likely be realized through rational combination strategies and context-specific scheduling rather than as monotherapy. Embedding SCD1 inhibition within multimodal ferroptosis-based regimens may therefore offer a way to overcome resistance in aggressive cancers while maintaining acceptable safety profiles.

## Conclusions

SCD1 has emerged as a central metabolic node through which diverse oncogenic, transcriptional, epigenetic, and microenvironmental signals converge to suppress ferroptosis across a spectrum of cancer types. Although the functional role of SCD1 in promoting MUFA biosynthesis and limiting lipid peroxidation is conserved, the regulatory logic is highly context-dependent: in oxidative environments such as the lung, hypoxia and redox stress bias upstream pathways toward SCD1 induction; in lipid-rich niches such as breast and ovarian tumors, extracellular lipid availability reinforces SCD1 activity; and in metabolically active organs such as the liver and pancreas, nutrient sensing and lipogenic transcriptional networks dominate SCD1 regulation. This review highlights how distinct metabolic constraints shape SCD1 regulation and ferroptosis resistance, providing a unifying framework that accommodates tumor heterogeneity and treatment-related adaptations.

From a translational perspective, multiple therapeutic opportunities arise from these insights. Rational combinations of SCD1 inhibition with ferroptosis inducers or conventional therapies appear promising, though systemic on-target toxicities remain a concern. Emerging strategies such as tumor-targeted delivery, prodrugs activated by tumor microenvironments, and adaptive/intermittent dosing schedules may mitigate toxicities while transiently lowering the ferroptosis threshold in malignant cells.
